# Resuscitative endovascular occlusion of the aorta restores cerebral metabolic markers of ischaemia induced by haemorrhagic shock

**DOI:** 10.1007/s00068-026-03147-y

**Published:** 2026-06-24

**Authors:** Sam Er Bader, A. Magnuson, C. Brorsson, G. Wallin, N. Löfgren, F. Löfgren, P-J Blind, M. Öman, M. Olivecrona

**Affiliations:** 1 Department of Surgery, Faculty of Medicine and Health, Örebro University, Örebro, Sweden; 2 Clinical Epidemiology and Biostatistics, School of Medical Sciences, Faculty of Medicine and Health, Örebro University, Örebro, Sweden; 3 Department of Surgical and Perioperative Sciences, Anaesthesia and Intensive Care, Umeå University, Umeå, Sweden; 4 Department of Diagnostics and Intervention, Umeå University, Umeå, Sweden; 5 Department of Neurosurgery, Faculty of Medicine and Health, Örebro University, Örebro, Sweden

**Keywords:** Resuscitative endovascular balloon occlusion of the aorta, REBOA, Haemorrhagic shock, Cerebral microdialysis, Metabolism, LPR, Ischemia, Aorta occlusion

## Abstract

**Background:**

Resuscitative endovascular balloon occlusion of the aorta (REBOA) is used as an adjunct in haemorrhagic shock (HS) to restore proximal perfusion. Its cerebral metabolic effects during haemorrhagic shock, particularly in the presence of elevated intracranial pressure (ICP), remain incompletely characterised.

**Methods:**

In a controlled porcine model, HS was induced by controlled bleeding to a mean arterial pressure of approximately 40 mmHg. Animals were allocated to a normal ICP group or an experimentally elevated ICP group. Total REBOA (zone 1) was applied for 90 min. Cerebral metabolism was assessed using intracerebral microdialysis with measurements of lactate, pyruvate, and lactate–pyruvate ratio (LPR). Cerebral haemodynamics and ICP were continuously monitored.

**Results:**

HS was associated with a statistically significant increase in LPR in both groups, indicating cerebral metabolic disturbance. Following aortic occlusion, LPR gradually decreased toward baseline in both groups. Animals with elevated ICP demonstrated a transient delay in metabolic normalisation during the early post-occlusion phase. Statistically significant differences between groups were limited to the first 10 min following occlusion. Overall metabolic trajectories were similar thereafter.

**Conclusions:**

Total REBOA restored cerebral metabolic markers of ischaemia during haemorrhagic shock, as reflected by normalisation of LPR, even in the presence of experimentally elevated ICP. These findings indicate acute metabolic recovery and so suggest that the use of tREBOA in the setting of elevated ICP is not contra indicated and can be a bridge to further treatment. Our findings do not demonstrate absence of tissue injury, or long-term neurological recovery. Further studies incorporating complementary physiological and structural outcome measures would be recommended.

## Background

In recent years, the use of Resuscitative Endovascular Balloon Occlusion of the Aorta (REBOA) as a less invasive procedure compared to aortic cross-clamping via thoracotomy [1] has increased as an adjunct resuscitative method and a bridge to damage control surgery, primarily in trauma settings involving haemodynamically unstable patients [2–4].

The main function of REBOA in haemorrhagic shock (HS) is to preserve the remaining blood to the upper part of the body, i.e. to the brain, the lungs, and the heart. Additionally, REBOA reduces ongoing bleeding distal to the occlusion until definitive haemostasis is achieved [5].

REBOA increases blood pressure proximal to the occlusion zone; consequently, it might be lifesaving and mitigates cerebral damage and neurological sequelae following the reduction of cerebral perfusion in patients with HS [6, 7].

Despite its haemodynamic benefits, concern remains regarding the cerebral consequences of abrupt proximal hypertension induced by aortic occlusion (AO). In particular, the use of total REBOA (tREBOA) has been questioned in the setting of traumatic brain injuries (TBI) associated with acute elevation in intracranial pressure (ICP), where supraphysiological arterial pressures could theoretically exacerbate intracranial hypertension or impair cerebral autoregulation [8–19].

Several animal studies in aortic and thoracic surgery have investigated the effects of AO on the brain using aortic cross-clamping. However, most of these studies were conducted on normovolemic animals [20, 21]. A few recent animal studies have focused on the cerebral effects of AO by REBOA in hypovolemic scenarios, primarily in trauma settings. These studies have mainly concentrated on haemodynamic and radiological changes in the brain [14, 22–25].

Cerebral microdialysis (CMD) is a validated method for monitoring chemical events and metabolic changes in cerebral tissue before they manifest in the blood [26]. Different neurochemical markers are used to detect early abnormal metabolic changes such as in the case of ischaemia, mitochondrial dysfunction, and cellular damage. Under normal conditions, glucose is the primary substrate for brain energy metabolism. The lactate/pyruvate ratio (LPR) indicates changes in the cellular redox state, and indicator for ischaemia [27–31].

To the best of our knowledge, the impact of thoracic AO in hypovolemic shock by REBOA on cerebral metabolism (CM) has not yet been described. However, we found two authors who have studied the metabolic changes in the spinal cord by inserting a microdialysis probe into the lumbar spinal cord during AO in normovoleumic pigs. They reported changes in energy-related metabolites reflecting considerable ischaemia in the spinal cord [32, 33].

The purpose of this paper is to fill the knowledge gap regarding the impact of total REBOA (tREBOA) on CM, in animals with HS.

The first aim is to describe the changes in CM during total AO during HS in animals with normal and elevated ICP, and the second aim is to study whether there is a difference between CM in the two groups. The null hypothesis is that there are more pronounced cerebral metabolic changes in the group with elevated ICP, indicating that tREBOA should not be used in individuals with suspected raised ICP.

## Material and methods

### Ethics

The study was approved by the Animal Experimental Ethics Committee at Umeå University, Sweden (A 32–19). And conducted in accordance with Directive 2010/63/EU on the protection of animals used for scientific purposes, and the *Guide for the Care and Use of Laboratory Animals*, National Research Council, Washington, DC, USA, 1996.

### Study overview

**Fig. 1:**
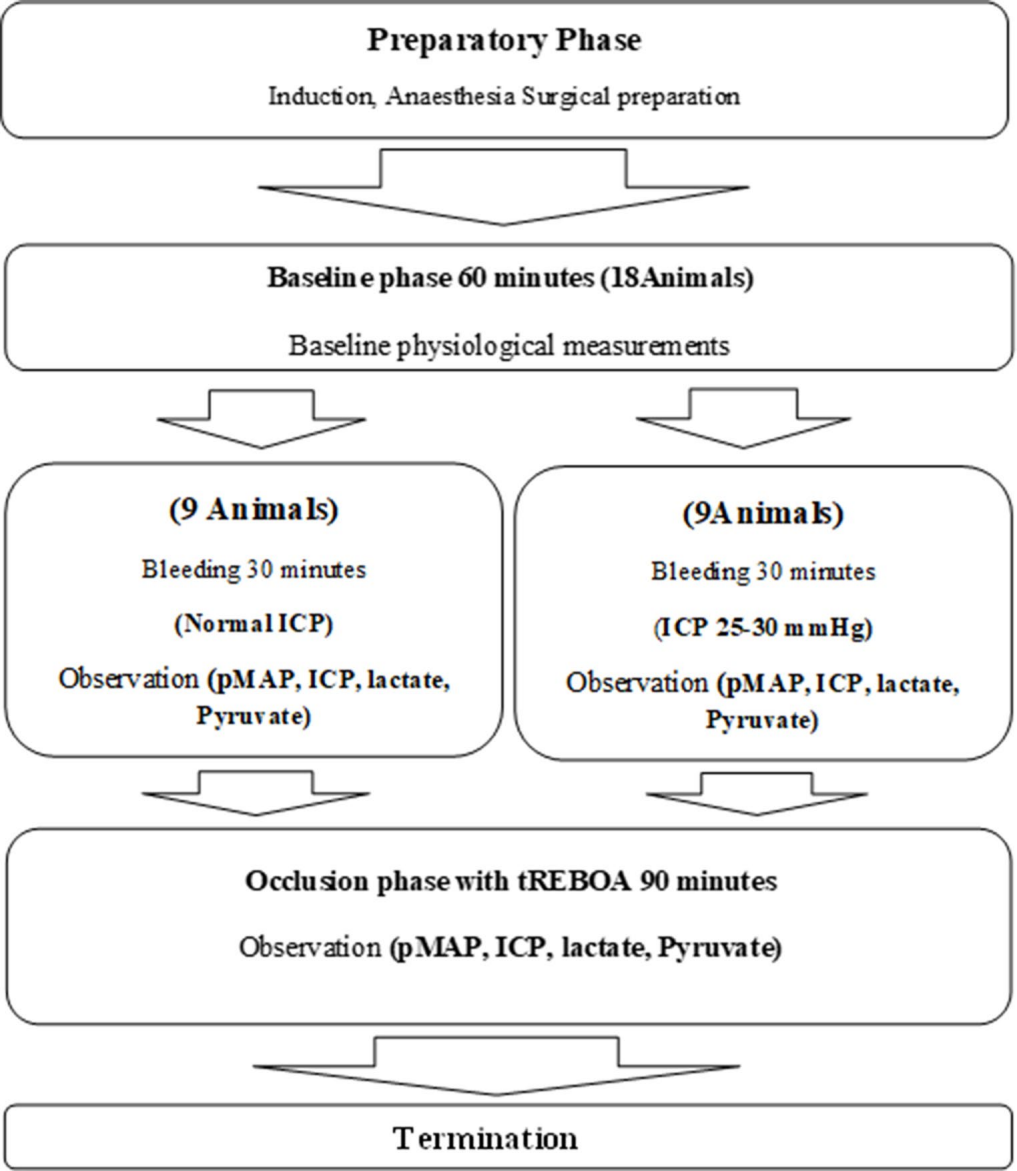
Experiment design

We studied two groups of animals with nine pigs in each, both males and females: The normal ICP group (NICPG), and the elevated ICP group (EICPG). The experiment comprised five phases illustrated in Fig. 1: the preparation phase, stabilisation phase, bleeding phase, occlusion phase, and lastly the termination phase (Fig. 1).

### Animal preparation

For detailed information about the general experimental setup and protocol, see Bader et al. [14].

#### Anaesthesia

The animals were sedated with ketamine and atropine sulphate, whereafter anaesthesia was started using sodium pentobarbital. Anaesthesia was maintained using fentanyl, midazolam, and sodium pentobarbital. After sedation, the animals were tracheostomised and mechanically ventilated. Ringer’s acetate was given as fluid maintaining a central venous pressure of 5–10 mmHg.

#### General monitoring and surgical preparation

Oxygen saturation was measured by pulse oximetry and heart rate was monitored by ECG. The CVP, PBP and the distal blood pressure in the femoral artery were monitored with the zero reference at heart level.

A REBOA was inserted via the right femoral artery. Confirmation of the balloon’s adequate placement in the aortic zone 1 was obtained under tactile guidance.

Saturation and ABP were documented every five minutes until the end of the experiment.

#### Cerebral monitoring

After anaesthesia and overall monitoring were established, the animal was placed in a prone position. The head was shaved, cleaned, and disinfected. The cranial bone was exposed with a paramedian incision 6 cm long on the left side. A 3 mm burr hole was drilled frontally. Next, the dura was opened sharply, and haemostasis secured by diathermy. A four-lumen bolt (H QFlow 500 Titanium, Hemedex, Waltham, MA, USA) was placed in the burr hole.

An intraparenchymal catheter measuring ICP and temperature (PSO-PTT, Sophysa, Orsay, France) was calibrated according to the manufacturer’s instructions, introduced through the bolt to a depth of 10 mm into the brain, and connected to an ICP monitor (PSO-4000 Pressio 2, Sophysa, Orsay, France). ICP was continuously measured and manually recorded every five minutes. CPP was calculated according to the formula CPP = MAP − ICP.

To simulate an acute epidural haematoma or epidural haematoma in EICPG, an extra 6 mm burr hole was drilled on the right side of the skull, where a Foley catheter (Ch12) was inserted into the epidural space. The balloon was inflated with saline to mimic an epidural haematoma.

### Microdialysis

A microdialysis catheter (70Brain Catheter, M dialysis AB, Stockholm, Sweden) was introduced through the bolt into the brain, aiming at an insertion depth of approximately 25 mm. The microdialysis probe was perfused with Perfusion Fluid CNS (M dialysis Stockholm, Sweden) using a microdialysis pump (CMA 107; CMA/ Microdialysis Stockholm, Sweden) at the flow rate of 2 µl/min. After at least one hour of stabilisation, microdialysis samples were taken at 5-minute intervals until the end of the experiment. Samples were analysed for glucose, lactate, and pyruvate using a CMA 600 Analyser (CMA Microdialysis AB, Stockholm, Sweden).

### Study design and experimental protocol

#### Stabilisation phase (60 minutes)

In a supine position with 15° table inclination, the animal was immobilised, keeping the head and heart on the same level; hence, the zero level of the PBP equals that of the ICP. Antibiotic prophylaxis with Cefuroxime 750 mg (MIP Pharma GmbH, Blieskastel, Germany) was given, and the 60-minutes stabilisation phase initiated.

#### Bleeding phase (30 minutes)

Blood volume for each animal was estimated at 8% of the animal’s body weight. Over 30 min 40% of the animal’s estimated blood volume was drained from the left femoral artery. The bleeding was calculated in ml/min according to the equation: Bleeding speed = Estimated blood volume / 30.

Intravenous infusion of 5000 IE Heparin (Heparin LEO Pharma AB; Malmö, Sweden) was administered before the bleeding started, and Adrenaline (Mylan, Canonsburg, Pennsylvania, USA) was given if needed to keep the MAP around 40 mmHg and to simulate the physiological stress response.

During this phase, the epidural Foley balloon in the EICPG was injected slowly with saline, aiming at an ICP of 25–30 mmHg.

#### Occlusion phase (90 minutes)

Once hypovolemia was achieved with MAP around 40 mmHg, the REBOA was inflated with saline (tREBOA) until ABP in the femoral artery disappeared (T = 0 min) and the tREBOA was kept inflated for 90 min.

After AO we administered intravenously 500 ml of 6% hydroxyethyl starch in sodium chloride (Voluven Fresenius Kabi, Homburg, Germany) to mimic prehospital treatment of haemorrhagic shock.

#### Termination

After 90 min of total AO, at a rate of 1 ml/min, the balloon was deflated, whereafter the animal was euthanized with a lethal intravenous injection of potassium chloride 40 mmol (Kaliumklorid; B. Braun Medical AB, Danderyd, Sweden) and sodium pentobarbital 400 mg.

### Statistics

The outcomes of pMAP, ICP, CPP, CL, CP and LPR were compared within and between study groups (EICPG, NICPG) at every 5- minute time point from − 30 until 90 min after AO with a random intercept linear mixed model. Study groups and time points and the interaction (group x time points) were used as fixed factors. All outcomes except pMAP and CPP were evaluated on the natural log scale, which showed better normal distribution assumptions for the standardised residuals. Sensitivity analyses were performed by excluding outliers if the standardised residual was greater than 3 (in absolute value). Association measures were mean differences with 95% confidence intervals (CI), and on the log scale, mean ratios (95% CI). A mean ratio of 1.20 interprets as the mean being 20% higher for exposed vs. unexposed. To reduce the risk of false-positive findings due to multiple testing because of many time points, a P-value below 0.01 was considered statistically significant. All analyses were performed with STATA release 17 (StataCorp, College Station, TX).

## Results

Eighteen pigs (thirteen female and five male) with a mean weight of 48.6 kg with (SD = 6.9) were included in statistical analysis.

### Proximal mean arterial pressure (pMAP)

During the bleeding phase in NICPG, pMAP showed a statistically significant decrease from initial mean 95 mmHg to baseline mean 49 mmHg before AO, mean difference 46 (95% CI 29–63; *P* < 0.01), then increased rapidly after AO, peaking 20 min later up to mean 129 mmHg, mean difference 80 (95% CI 63–97; *P* < 0.01), and remaining elevated throughout the experiment.

A similar pattern was observed in the EICPG; however, pMAP reached its peak of mean 168 mmHg 15 min post-AO, mean difference 122 (95% CI 105–139; *P* < 0.01).

**Fig. 2:**
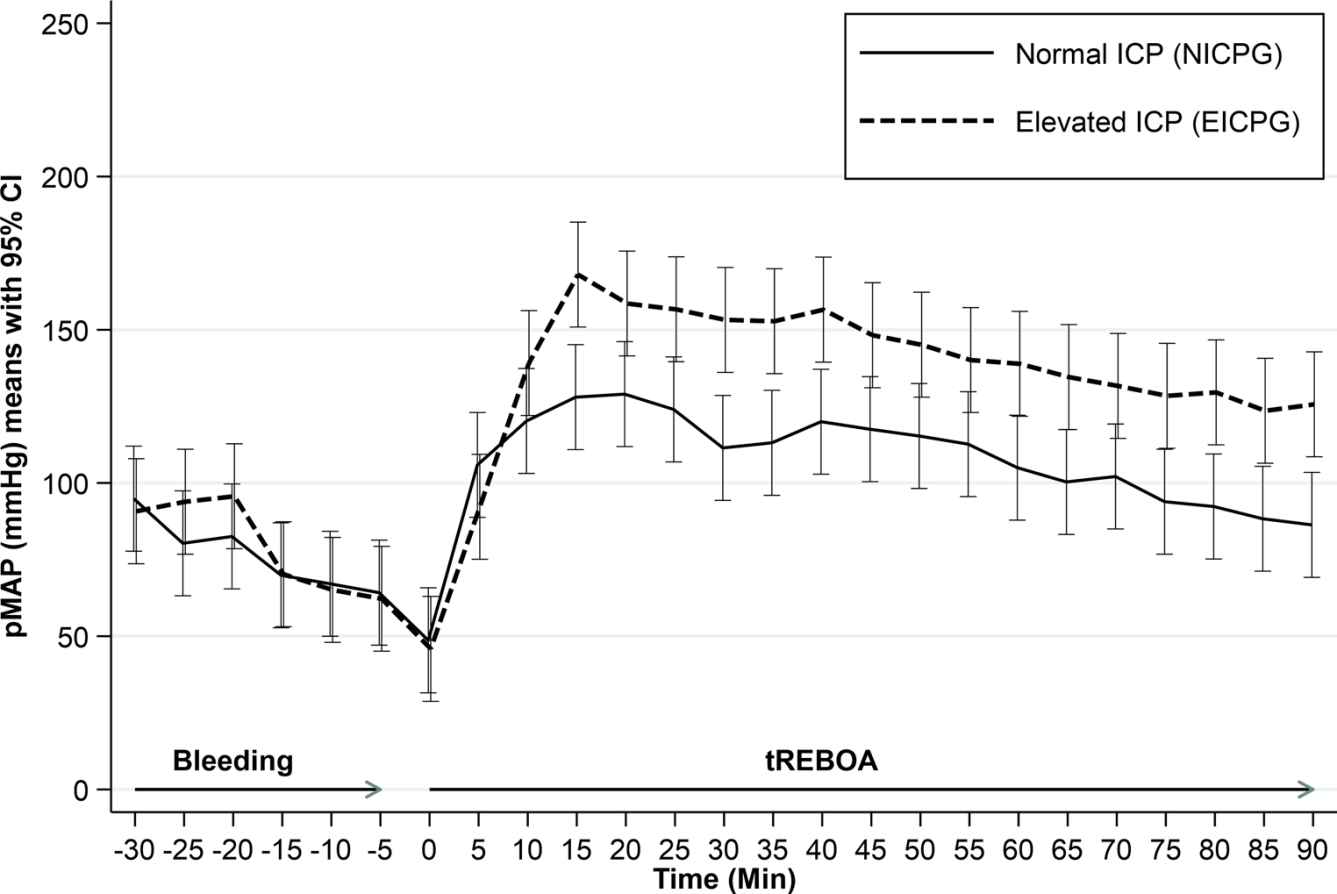
Mean Arterial Pressure (MAP)

No statistically significant difference in pMAP was observed between groups during the bleeding phase. However, the pMAP in the EICPG was statistically significantly higher than in the NICPG from 15 min post-AO onward (Fig. 2).

### Intracranial pressure (ICP)

In the NICPG, ICP remained stable with a mean of around 15 mmHg throughout the experiment.

In the EICPG, inflation of the epidural balloon led to a significant rise in ICP from mean 17 mmHg to mean 29 mmHg prior to AO, mean difference 12 (95% CI 0.48–0.71; *P* < 0.01). After AO, ICP further increased, reaching a maximum of mean 38 mmHg at 5 min post-AO, though this was not statistically significant compared to pre-AO baseline, mean difference 9 (95% CI 1.06–1.57; *P* > 0.01).

**Fig. 3:**
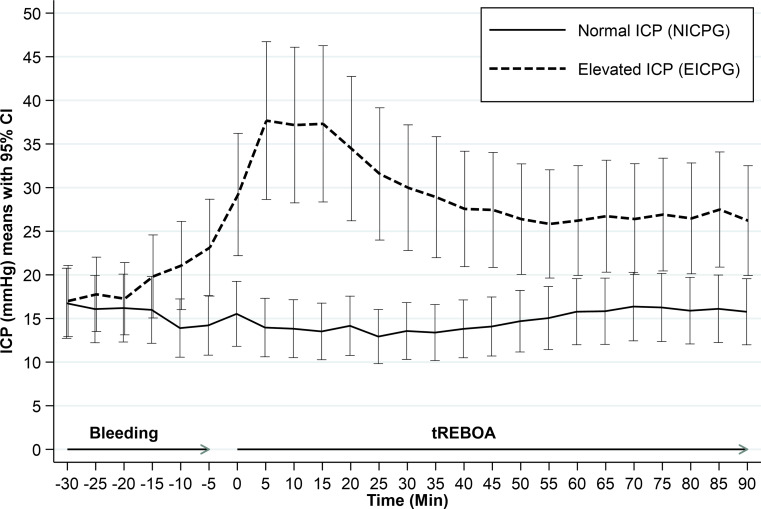
Intracranial Pressure (ICP) vs Time

ICP was statistically significantly higher in EICPG than in NICPG from 5 min before AO until the end of the experiment (Fig. 3).

### Cerebral perfusion pressure (CPP)

In the NICPG, CPP declined from mean 78 mmHg to mean 32 mmHg during the bleeding phase, mean difference 46 (95% CI 28–63; *P* < 0.01), then rose rapidly after AO, peaking at mean 115 mmHg after 20 min, mean difference 83 (95% CI 65–100; *P* < 0.01). It remained statistically significantly elevated compared to pre-AO baseline for the rest of the experiment.

In the EICPG, CPP dropped from mean 73 mmHg to mean 16 mmHg pre-AO, mean difference 57 (95% CI 40–75; *P* < 0.01). and rose to a peak of mean 124 mmHg 15 min post-AO, mean difference 108 (95% CI 91–126; *P* < 0.01)., staying statistically significantly above baseline thereafter.

**Fig. 4:**
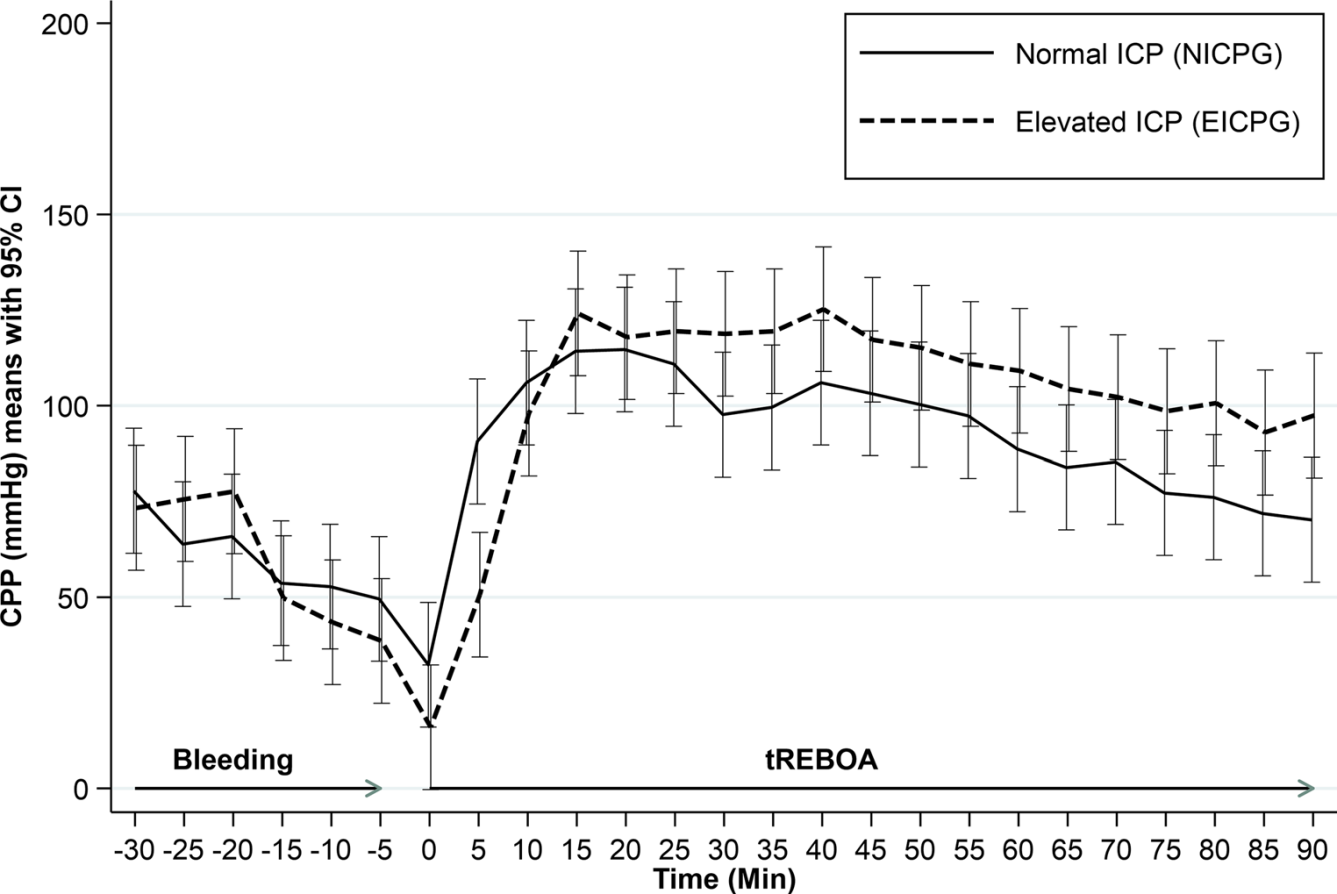
Cerebral Perfusion Pressure (CPP) vs Time

There was no statistically significant difference in CPP between the groups, except at one time point (5 min post-AO), where EICPG had significantly lower CPP with mean 51mmHg than NICPG with mean 91mmHg, mean difference 40 (95% CI -63-17; *P* < 0.01) (Fig. 4).

### Cerebral lactate (CL)

In the NICPG, CL gradually increased during the bleeding phase from mean 0.71 mmol/L to mean 1.13 mmol/L pre-AO, mean difference 0.42 (95% CI -0.46-0.85; *P* < 0.01), and continued to rise slowly during the occlusion phase until the end of the experiment, without statistically significance.

In the EICPG, CL started to increase earlier than in NICPG and reached statistically significant elevated levels from 55 min into the timeline.

**Fig. 5:**
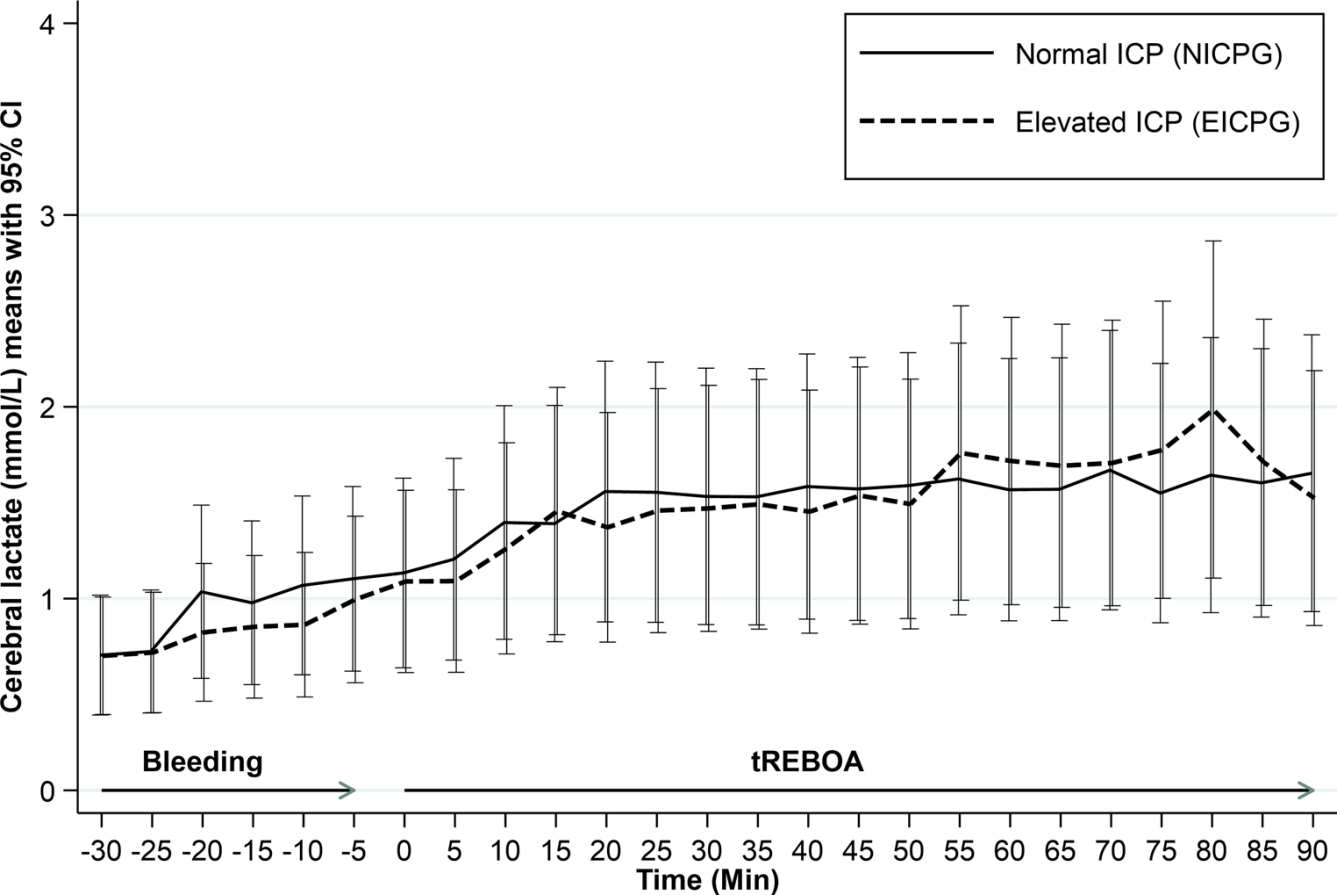
Cerebral Lactate vs Time

No statistically significant differences in CL were observed between the groups throughout the experiment (Fig. 5).

### Cerebral pyruvate (CP)

In the NICPG, CP increased statistically significantly at 20 min post-AO from mean 24 µmol/L to mean 42 µmol/L, mean difference 18 (95% CI 1.2–2.6; *P* < 0.01), and remained elevated.

In the EICPG, CP remained stable during bleeding but increased statistically significantly 15 min after AO from mean 9 µmol/L to mean 20 µmol/L, mean difference 11 (95% CI 1.5-3; *P* < 0.01), and stayed elevated.

**Fig. 6:**
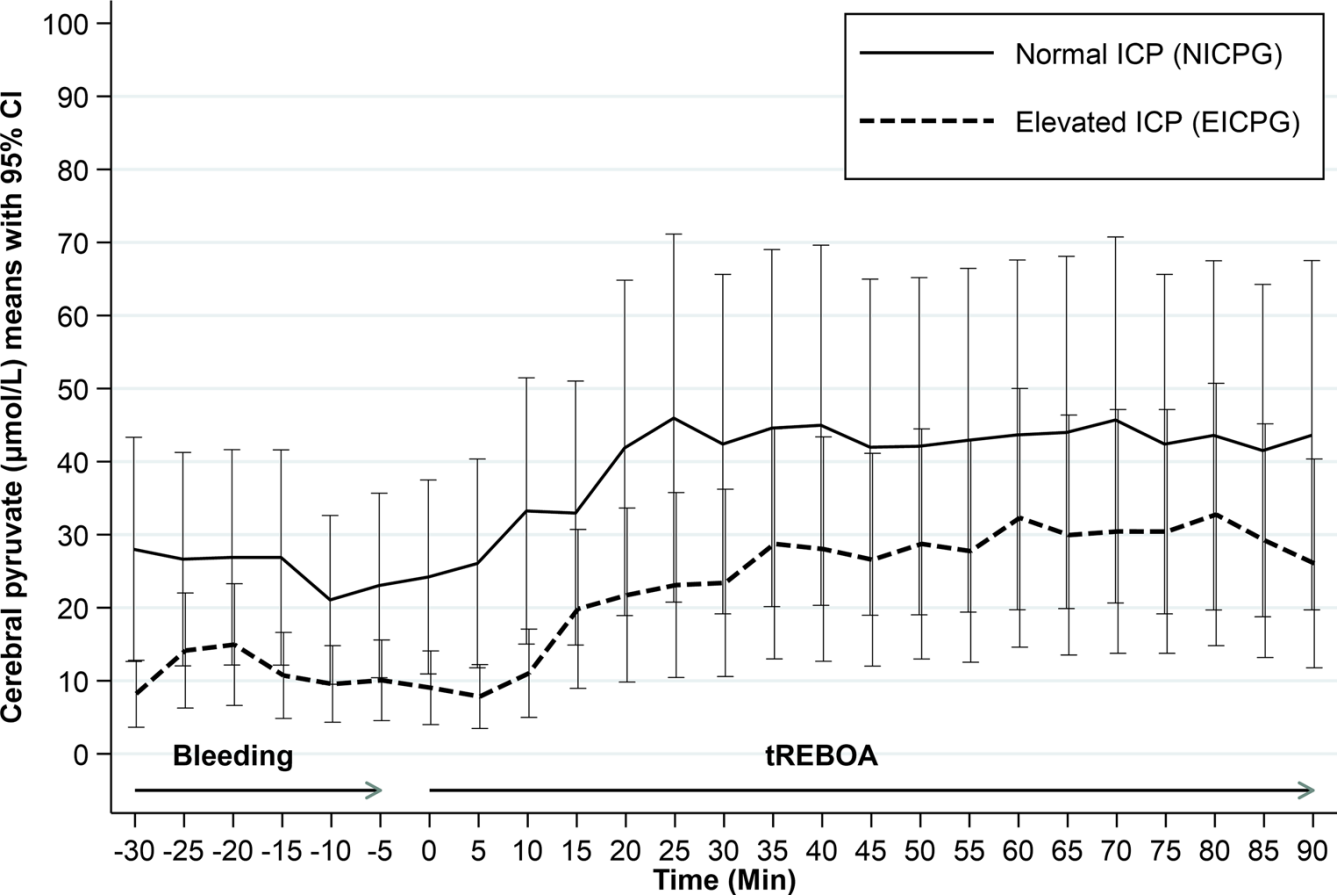
Cerebral Pyruvate vs Time

Initial CP levels were statistically significantly lower in the EICPG compared to NICPG (*P* < 0.01) and 5 and 10 min after AO (*P* < 0.01). However, in the sensitivity analysis, excluding outliers, the differences at 5 and 10 min after AO were no longer statistically significant (Fig. 6).

### Cerebral lactate pyruvate ratio (LPR)

In the NICPG, LPR rose from mean of 26 to 47 during bleeding, mean difference 21 (95% CI 0.35–0.85; *P* < 0.01), and gradually decreased after AO, reaching a low mean level of 34 by 25 min post-AO, mean difference 13 (95% CI -0.47-1.1; *P* < 0.01), but never reached statistically significantly low levels compared to pre-AO until the end of the experiment.

In the EICPG, LPR also rose during bleeding phase from mean 56 to mean 121 prior AO, mean difference 65 (95% CI -0.29-0.72; *P* < 0.01), and increased further 5 min post-AO before gradually declining and remained statistically significantly low compared to pre-AO.

**Fig. 7:**
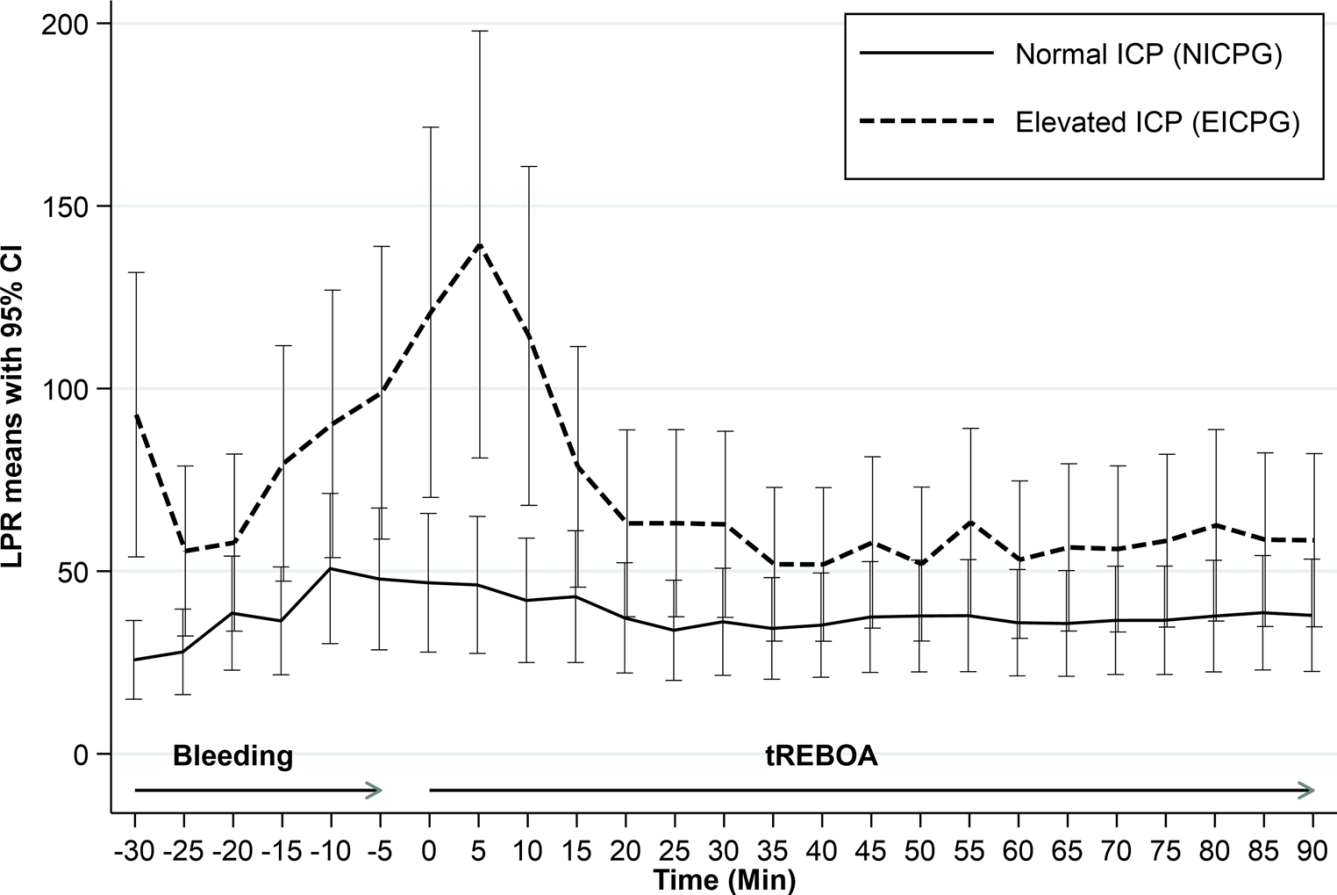
Lactate Pyruvate Ratio (LPR) vs Time

Statistically significant differences between groups were found during the first 10 min post-AO only (Fig. 7).

## Discussion

Although the human brain accounts for only about 2% of total body weight, it receives nearly 20% of the cardiac output and body energy production. Due to its limited energy reserves, even brief interruptions in CBF can rapidly result in severe neurological impairment [34]. The cerebral cytoplasmic redox state, expressed by the lactate-to-pyruvate ratio (LPR), serves as a sensitive indicator of mitochondrial oxidative metabolism [27–30, 35, 36].

In this experimental model, we demonstrated at the cellular level that AO using tREBOA is an effective resuscitative strategy in uncontrolled HS.

During HS in the NICPG, LPR increased slightly due to elevated lactate and decreased pyruvate levels but returned to baseline following AO, indicating reversibility of cerebral metabolic disturbance. The same pattern was observed in EICPG, however, LPR reached higher values with about a 20-minute delay before returning to baseline.

We observed a brief but statistically significant difference in LPR between groups during the first 10 min after occlusion. This corresponded to a statistically significant difference in CPP during the same period. During the first 10 min of AO, CPP decreased to critically low levels in EICPG, which may indicate impaired CA [14].

The abrupt increase in pMAP induced by AO did not significantly affect cerebral metabolism in the NICPG. However, in EICPG, a transient and reversible metabolic disturbance was observed, evidenced by an LPR peak following the supraphysiological pMAP (129 mmHg) induced by AO.

Our haemodynamic findings are consistent with previous reports that described comparable effects of AO and tREBOA on MAP, ICP, and CPP in HS [14, 22–25]. Normalisation of LPR indicates restoration of cellular redox balance but does not provide evidence regarding tissue integrity, blood–brain barrier (BBB) function, or neurological outcome.

In trauma, HS frequently coexists with elevated ICP. Approximately 80% of trauma-related deaths are attributed to uncontrolled haemorrhage and 4% to TBI [37–40]. The coexistence of these two conditions nearly doubles morbidity and mortality compared to HS alone [41, 42].

Time is a critical factor in trauma management, especially in haemodynamically unstable patients. Fluid resuscitation alone may be insufficient to restore cardiac output and cerebral perfusion. Achieving distal control of bleeding can also be challenging in cases of massive haemorrhage.

Inflation of the tREBOA balloon in aortic zone 1 divides the arterial system into two distinct circulatory compartments: a proximal (upper body) circulation and a distal (lower body) circulation. However, minimal collateral flow persists through arterial connections between these two regions [43].

Our animal model replicates two clinical trauma scenarios: First, NICPG representing HS scenario resuscitated by tREBOA with a target mean arterial pressure (MAP) of approximately 40 mmHg, as recommended by the European Society for Intensive Care Medicine [44]. Second, EICPG representing HS scenario associated with acute ICP (25–30 mmHg), following the method described by Timaru-Kast et al. This model reliably induces reproducible ICP elevation and associated cerebral energy metabolism alterations similar to those observed in TBI [45]. The acute ICP elevation occurs in 7–23% of the total TBI cases, 8–15% caused by acute subdural haematoma and 2–8% due to epidural haematoma [46–48].

### Normal ICP group (NICPG)

#### Bleeding phase

In NICPG, LPR increased significantly between the beginning and end of the bleeding phase (*P* = 0.01), indicating cerebral metabolic disturbance. Similar findings were reported by Jakobsen et al., who observed a reversible cerebral ischemia with increased LPR in anesthetised pigs during HS (MAP 40 mmHg for 60 min) resuscitated with autologous blood, although LPR decreased after resuscitation, it remained elevated above baseline [49]. In another experiment for Jakobsen R et al. published in 2016 shown that prolonged (90 min) and severe hypotension (MAP 40 mmHg) results in irreversible metabolic perturbation evaluated by CMD [31].

Cerebral ischaemia typically results from reduced CPP or elevated ICP, leading to well-known metabolic disturbances with low oxygen and low pyruvate concentration. Oxygen deprivation during ischaemia causes an immediate rise in cytoplasmic and interstitial LPR due to a shift in the lactate dehydrogenase equilibrium [27, 50].

The observed LPR elevation coincided with MAP falling below 50 mmHg, consistent with transient impairment of CA and reduced CBF [14].

CA maintains constant CBF across a broad CPP range, defined as MAP minus ICP. With normal ICP (5–10 mmHg), CBF remains stable up to MAP values of approximately 120 mmHg. Once CPP decreases below 50 mmHg, autoregulation fails, resulting in progressive cerebral dysfunction [51]. When blood flows decline to 25–30 mL/100 g/min, electroencephalographic changes occur along with alterations in consciousness. With further decreasing below 20 mL/100 g/min, electroencephalogram become isoelectric, and neurons switch to anaerobic metabolism. At 10–12 mL/100 g/min, neurotransmission stops, sodium-potassium pumps fail, and cytotoxic oedema develops. Finaly, at 6–10 mL/100 g/min, cerebral tissue dies [34, 52].

#### Occlusion phase

After 20 min of tREBOA resuscitation, LPR gradually declined and stabilised, simultaneous to increases in CPP and MAP. This decline indicates the recovery of cerebral metabolism and reversal of ischaemia; however, tissue injury can’t be excluded. When cerebral oxygenation is promptly restored, LPR typically returns to near-normal levels. Lactate and pyruvate, being water-soluble, quickly equilibrate across the BBB and cell membranes [50].

#### Elevated ICP group (EICPG)

##### Bleeding phase

During haemorrhage, LPR rose exponentially, reaching significantly higher levels than in NICPG, indicating more pronounced cerebral metabolic disturbance. The increase in LPR corresponded with critical reductions in CPP (16 mmHg), aggravated by artificially elevated ICP.

According to the Monro–Kellie doctrine, intracranial volume is constant, comprising blood brain tissue, and cerebrospinal fluid. Disruption of this equilibrium through haemorrhage, ischaemia, hydrocephalus, or reperfusion injury raises ICP and can lead to reduced CPP, cerebral ischaemia, or herniation [53–55].

In intact autoregulation, hypotension elevates ICP, whereas hypertension may have minimal impact. However, in cases of impaired autoregulation, ICP fluctuates directly with blood pressure [56].

Increased ICP without compensatory hypertension reduces cerebral perfusion and may trigger the Cushing reflex, a physiological triad of hypertension, bradycardia, and irregular respiration in response to ICP > 25 mmHg [57].

### Occlusion phase

LPR was already increased by the end of bleeding phase. After AO, a further increase occurred after AO, for about 5 min, then decreased significantly after 20 min, returning to baseline within 30 min. This transient rise suggests reversible metabolic disturbance.

The supraphysiologic arterial pressure up to 168 mmHg in this group can be explained by a double mechanism: The effect of AO itself on cerebral blood volume and alteration of autoregulation, and by Cushing reflex due to ICP increasing over 25 mmHg [14, 57].

In a study by Bader et al., the authors showed that total AO by tREBOA induces a reversible alteration of CA with positive values of the Modified-Long Pressure Reactivity Index (mL-PRx) [14].

Previous studies demonstrated that acute hypertension caused by descending AO can elevate CBF, cause vasodilation, and disrupt the BBB [58–65].

Other experimental studies about the metabolic effects of acute hypertension induced by angiotensin on the brain showed that LPR was not affected but produced multifocal BBB leakage of Evans blue albumin [66, 67].

Autoregulation remains the brain’s primary protective mechanism against acute hypertension, adjusting cerebrovascular resistance to maintain stable flow [68, 69]. Yet its capacity diminishes under sudden or extreme pressure changes [70]. Mild increases in ICP activate astrocyte-mediated sympathetic responses to preserve perfusion whereas larger increases trigger the Cushing reflex [71–73].

Thus, elevated ICP during hypertension might further impair cerebral perfusion due to dysfunction of autoregulatory and baroreflex mechanisms [74–76]. Moreover, the BBB might play a key role in regulating cerebral volume, as its low permeability to solutes such as sodium and chloride makes it a critical determinant of brain homeostasis [77].

The transient nature of group differences in LPR suggests that the cerebral metabolic response to restored perfusion is largely similar regardless of ICP status once proximal haemodynamics are stabilised. This finding is physiologically plausible and supports the interpretation of the study as providing mechanistic insight rather than a paradigm-shifting effect.

## Limitations

Several limitations should be acknowledged. First, cerebral microdialysis provides local metabolic information and may not reflect regional or global cerebral metabolism. Second, anaesthesia, heparinisation, and experimental conditions may also have influenced cerebral metabolism. Third, the elevated ICP model represents acute intracranial hypertension rather than a parenchymal traumatic brain injury. Fourth, while LPR indicates metabolic stress, normalization does not necessarily reflect resolution of tissue injury, moreover, the study did not include complementary measures of cerebral blood flow, brain tissue oxygenation, structural injury, or blood–brain barrier integrity. Finaly, 90 min of total occlusion was longer than recommended in the clinical scenarios, and the observation period was limited and did not allow assessment of delayed or reperfusion-related injuries.

## Conclusion

Total REBOA restored cerebral metabolic markers of ischaemia during haemorrhagic shock, as reflected by normalisation of LPR, even in the presence of experimentally elevated ICP. These findings indicate acute metabolic recovery and so suggest that the use of tREBOA in the setting of elevated ICP is not contra indicated and can be a bridge to further treatment. Our findings do not demonstrate absence of tissue injury, or long-term neurological recovery. Further studies incorporating complementary physiological and structural outcome measures would be recommended.

## Data Availability

No datasets were generated or analysed during the current study.

## Abbreviations

AO: Aortic Occlusion
BBB: Blood-Brain Barrier
CA: Cerebral Autoregulation
CBF: Cerebral Blood Flow
CH: Cerebral Hemodynamics
CL: Cerebral Lactate
CM: Cerebral Metabolism
CMD: Cerebral Microdialysis
CP: Cerebral Pyruvate
CPP: Cerebral Perfusion Pressure
EICPG: Elevated Intracranial Pressure Group
HS: Haemorrhagic Shock
ICP: Intracranial Pressure
LPR: Lactate Pyruvate Ratio
MAP: Mean Arterial Pressure
NICPG: Normal Intracranial Pressure Group
pMAP: Proximal Mean Arterial Pressure
PRX: Pressure Reactivity Index
REBOA: Resuscitative Endovascular Balloon Occlusion of the Aorta
TBI: Traumatic Brain Injury
tREBOA: Total Resuscitative Endovascular Balloon Occlusion of the Aorta
